# Using Mobile Devices in Teaching Large University Classes: How Does It Affect Exam Success?

**DOI:** 10.3389/fpsyg.2020.01363

**Published:** 2020-06-30

**Authors:** Tommaso Feraco, Nicole Casali, Carla Tortora, Cecilia Dal Bon, Donatella Accarrino, Chiara Meneghetti

**Affiliations:** ^1^Department of General Psychology, University of Padua, Padua, Italy; ^2^Pentathlon Srl, Naples, Italy; ^3^Digitial Learning Center, University of Padua, Padua, Italy

**Keywords:** student response systems, technology-enhanced teaching, innovative teaching, academic achievement, large classes, Italian university

## Abstract

The aim of this study was to examine the role of mobile-based student response systems in teaching to improve university students’ academic outcomes. Mobile devices can be useful tools for conveying content to large classes, with a potential impact on academic outcomes. This study involved a total of 294 undergraduates taking a psychology course. The course involved lessons in the classroom, which included answering quizzes (quiz activities) and activities such as preparing reports and laboratory experiences (out-of-class activities). Quizzes were administered using a mobile technology system. Data were collected on the motivational beliefs (theory of intelligence) and self-regulated learning strategies of students who voluntarily completed the online questionnaires. The results of the linear models showed that using the quizzes positively affected the performance in the final exams (involving closed and open questions). The same was true for the out-of-class activities. The motivation and strategy scores correlated moderately with out-of-class activities, but not with quiz activities. These results offer insight on the use of technology during lessons and other course-related activities to promote academic achievement.

## Introduction

One of the main aims of higher education systems is to enable students to learn effectively. Teaching modalities and practices can play an important part in academic achievement throughout a student’s university career (Caird and Lane, 2015). Among several teaching approaches, technology systems based on mobile devices are potentially powerful learning tools. The European Commission is increasingly advocating integration between technology and teaching to increase efficiency in higher education systems (e.g., Digital Education Action Plan; European Commission, 2018).

The interest in innovative teaching methods is nourished by a growing body of research showing the benefits of exploiting technologies during lectures at a university, as well as in various other higher-education settings, to learning and learning-related outcomes (Sung et al., 2016). These benefits are particularly important in contexts where university classes are large and/or attendance is not mandatory, making it more challenging for lecturers to encourage students’ participation in class (Hunsu et al., 2016).

Among technology systems, mobile-based student response systems (SRS, also known as audience/classroom response systems or “clickers”) are attracting particular attention as a way for teachers to interact actively with students (Kay and LeSage, 2009). SRS allow students to answer closed (multiple-choice or true/false) questions displayed on a screen during lessons by pressing a button on a keypad, mobile phone screen, or web-based interface (hence the name “clickers”) and to receive immediate feedback. The literature on this topic is flourishing, with three recent meta-analyses on the influence of these methods on various learning domains, such as academic performance (Castillo-Manzano et al., 2016; Sung et al., 2016), and on related learning outcomes like engagement, which means students’ involvement or participation in class (Hunsu et al., 2016). On average, SRS have shown a small-to-medium positive effect on achievement [0.21 < *g* < 0.37 in Hunsu et al. (2016), Castillo-Manzano et al. (2016), and Sung et al. (2016)] and students’ participation in class (*g* = 0.19, Hunsu et al., 2016).

The positive effect of quizzes, administered through a SRS, on academic performance can be due to optimizing the learning process and stimulating cognitive mechanisms involved in learning, such as testing effect and feedback. The testing effect (e.g., Roediger and Karpicke, 2006; Roediger and Butler, 2011) is a well-known cognitive phenomenon according to which taking practice tests promotes learning and long-term retention better than other study strategies, such as re-reading. A recent meta-analysis (118 studies; Adesope et al., 2017) found that testing positively influences achievement (*g* = 0.60 at university level) and is more effective than re-reading (*g* = 0.51). Interestingly, the testing effect was not moderated by the presence or the absence of feedback, although there is evidence of immediate feedback yielding greater retention benefits than delayed feedback (Butler et al., 2007), and corrective feedback seems to enhance the testing effect (Rowland, 2014). The efficacy of SRS quizzes on achievement may therefore be explained by both testing effect and immediate feedback. Most studies on testing effect have focused on regular-sized classrooms, with fewer than 100 students (e.g., Roediger and Butler, 2011), while none—to our knowledge—have specifically investigated whether the testing effect is preserved in large classrooms. A moderator analysis from the meta-analysis by Hunsu et al. (2016) on SRS and achievement suggests that the positive effect of using clickers on performance declines with increasing classroom size (small effect with 100–200 students, *g* = 0.10, compared to *g* = 0.58 with 21–30 students). The testing effect (believed to explain the efficacy of SRS quizzes) may therefore be reduced in large university classes, too.

The present study aims to analyze the effect on academic performance of using interactive teaching practices in large classes where attendance is not mandatory.

Concerning interactive technologies, among several SRS-based quizzes available in the market (e.g., Kahoot!, Socrative, and Learning Catalytics), Top Hat^1^ is one of the systems most often used in higher education nowadays. The Italian university where our study was conducted has been supporting its use for numerous courses (182 courses, involving a total of 12,612 students in 2018–2019). Top Hat has proved particularly effective and been much appreciated by students (Neilson et al., 2016; Kawash and Collier, 2018). It assures anonymity, allowing students to receive feedback without feeling exposed to the judgment of peers or teachers (Salzer, 2018). It reduces the use of smartphones and laptops in class for distraction purposes (Rozgonjuk et al., 2018) and enhances engagement in lectures (Kawash and Collier, 2018). Top Hat is also able to integrate quizzes (and other activities) with lecture content (such as PowerPoint presentations) with more refined features than other SRS systems, making quizzes a complementary part of the lesson in real time.

The present study specifically examined whether using SRS-based quizzes during university lectures can positively affect the final exam performance. If answering quizzes helps students to memorize information better than by just listening to lectures, as seen in studies on the testing effect (Roediger and Karpicke, 2006; Roediger and Butler, 2011), and providing immediate feedback on their answers can consolidate their learning (Rowland, 2014), then we can expect the quiz activities during lessons to have an impact on the final exam performance.

The use of quizzes together with other teaching methods was examined, given the potential efficacy of combining several types of activities (Sung et al., 2016). Among the various potentially activating teaching methods, we opted for out-of-class activities that further elaborated on the course content, such as laboratory experiences and preparing reports. These activities are designed to make learning of the course material more effective, with an impact on academic performance. To our knowledge, the effect of such complementary activities on academic performance has yet to be thoroughly examined, although there is some evidence supporting their usefulness (Heng, 2014; Ko et al., 2015; Ribeiro et al., 2019). For instance, Heng (2014) found a positive contribution of out-of-class educational activities (e.g., time spent on course-related tasks, reading before lectures) to undergraduates’ academic performance, explaining around 20% of the variance in achievement (exam performance).

Participation in quiz activities and out-of-class activities was not mandatory, and a student’s decision to exploit these opportunities might be a sign of more active engagement. Students’ participation in class is an indicator of behavioral engagement, part of a multi-dimensional construct that includes cognitive (e.g., the cognitive aspects involved and the ability to regulate them) and affective (e.g., the type of emotion and motivation experienced) components [see Bond et al. (2020) for a review]. Therefore, since strategic and motivational aspects are considered as proxies of cognitive and emotional engagement (Bond et al., 2020), we examined whether students’ self-regulated learning strategies (e.g., Zimmerman, 2000) and motivational beliefs, such as incremental theories of intelligence (Dweck, 2000), are related to their use of quizzes and out-of-class activities. We also investigated whether study strategies and motivation were positively associated with final exam performance [as seen by Lei et al. (2018), Mega et al. (2014), and Richardson et al. (2012), for instance]. To achieve this purpose, we invited students to answer online questionnaires.

## Method

### Participants

The sample consisted of 294 undergraduates (72 males; age range, 20–25 years) from two cohorts (153 from the 2018 cohort and 141 from the 2019 cohort) enrolled on a psychology course for a Bachelor’s Degree in Psychology (for which 150 students had enrolled). The course lasts 42 h (11 weeks, 21 lessons, with two weekly sessions lasting 90 min each). Since we could not plan the number of participants in advance, we ran a retrospective power analysis *via* simulation on the number of participants collected to test the reliability of our results (Altoè et al., 2020). Hypothesizing a plausible correlation of 0.30 between exam results and the use of quizzes and out-of-class activities (Castillo-Manzano et al., 2016; Hunsu et al., 2016; Sung et al., 2016; Lei et al., 2018), 294 participants yielded a power of 0.84, with a plausible magnitude error of 0.72 and no sign error. The study was approved by the Ethics Committee for Research in Psychology at the University of Padova (N. 3519).

### Materials

#### Activities

##### Quiz activities

Using Top Hat, a total of seven quiz sessions were planned, with three to 12 questions each (*M* = 6.57, SD = 3.26), consisting of multiple-choice (77%), true/false (18%), or click-on-target (touching part of a figure, 5%) questions. During the lessons, the teacher launched Top Hat (using the teacher’s credentials), and the students accessed the quiz on their own devices (smartphones, tablets, and laptops) using their academic credentials. Each question was shown on their screens together with the possible answers. After they had selected their answers, the right answer and the collective frequencies of right, wrong, and n/a answers were shown. All students’ answers were recorded. The number of sessions where a student answered at least one question was used as a final measure (maximum: seven).

##### Out-of-class activities

Three other types of activity were proposed: (1) prepare a group presentation on a topic related to the course content (based on research or reviews published in scientific journals) and present it in class (group work), (2) participate in one or two laboratory studies (lab experiences), and (3) describe a person’s psychological profile starting from their scores in personality questionnaires and then have the resulting description blind-judged by two classmates on clarity and correctness, all under the teacher’s supervision (case report). These activities were presented during the first lesson and were not mandatory, but participation was rewarded with bonus points (one point for group work, one point for the first lab experience, one point for the second lab experience, and one point for case reports) on the students’ final exam score. The number of activities that the students engaged in was counted as a final measure (maximum: four).

#### Motivation and Strategy Questionnaires

##### Theory of Intelligence Questionnaire

This Theory of Intelligence Questionnaire (TIQ;Dweck, 2000; De Beni et al., 2014) consists of eight items that measure incremental and static theories of intelligence, i.e., beliefs about whether or not one’s own intelligence can be modified (e.g., “You can learn new things, but you can’t change your intelligence”). The answers are given on a six-point Likert scale (1 = “completely agree” to 6 = “completely disagree”). The internal consistency is good (α = 0.88).

##### Self-Regulated Strategy Questionnaire

This Self-Regulated Strategy Questionnaire (SRSQ; De Beni et al., 2014) contains 50 items that assess self-regulated learning strategies, i.e., organization, elaboration, self-evaluation, preparing for exams, and metacognition (e.g., “I like to think about how my mind works”). The answers are given on a five-point Likert scale (1 = “completely agree” to 5 = “completely disagree”). The internal consistency is good (α = 0.76).

#### Exam Performance

##### Exam

The exam consists of two parts: 30 closed questions (one point for each correct answer, 0 point if left blank, and minus one for wrong answers; maximum = 30) that differ from those used in the quizzes, plus four mandatory open questions (7.5 points for each answer; total 30). The exam also includes a list of five optional open questions on specific course content that can earn up to six bonus points. The questions are selected from a large pool already available and double-checked for clarity by other professors. The mean score for the two parts is calculated as the student’s exam performance. When students obtain a raw score ≥33, they are awarded the maximum possible score of 30 with honors. The data were only collected for the students’ first exam attempt. The bonus points awarded for the out-of-class activities were not considered in the analyses.

### Procedure

The psychology course was held in the first semester of the academic year, from October to December. During the first lesson, the teacher (the last author) presented quiz activities and out-of-class activities, inviting students to register in the SRS. She explained that the impact of their use on exam performance would be the object of a study. All the students signed the consent form. The quizzes were administered in one of the two weekly lessons using the Top Hat system, which was integrated with the use of slides, presenting the course content, that were interspersed with the quizzes. After each question was answered, the feedback was projected on the students’ personal devices and on the classroom projector screen, indicating the percentages of the respondents’ right and wrong answers. Each 90-min lesson involving quizzes included one to three sets of mostly multiple-choice questions (3 to 12 questions in all) presented at the beginning, in the middle, and/or at the end of the lesson. Each question took up 2 to 5 min, considering the time taken to answer and then to project the feedback (i.e., 1 min per question) and the time spent discussing the answers. The time spent in each lesson on the quizzes varied from around 6–15 to 24–60 min. The students in class completed the quizzes without any difficulty, although technical problems could sporadically occur (when logging out of the system). During the course, the students were also invited to complete the SRSQ and the TIQ using the university online platform (Moodle). This was not mandatory and was done by the students in their own time. At the end of the course, the students could decide when to take the exam on one of five dates in January, February, June, July, or September.

## Results

### Preliminary Analysis

Of the 294 students, 187 participated in both the quiz and the out-of-class activities (63.60%), 55 were only involved in the latter (18.71%), and 52 in neither (17.69%); none of the students participated in quiz activities only. Concerning the out-of-class activities, of the 242 students taking part, 87 completed all four activities, 34 only produced a case report, 21 only participated in the group work, and only eight were involved in lab experiences, while 92 engaged in two or three different activities. The results of a *t*-test for the independent samples showed that the students who did not participate in any of these activities had lower final exam performance than those who did [*t* (292) = 3.81, *p* < 0.001; exam score: no activity, *N* = 52; *M* = 20.01; SD = 4.98; participating group, *N* = 242, *M* = 22.86, SD = 4.86). Table 1 shows the means, standard deviations, and correlations between all measures.

**TABLE 1:** Means, standard deviations, and correlations for all the variables included in the study.

	M	SD	1	2	3	4	5
Exam performance	22.36	5.00	–				
Quiz activities	2.31	1.42	0.30***	–			
Out-of-class activities	2.38	2.48	0.32***	0.45***	–		
Theory of Intelligence Questionnaire	28.19	2.31	0.11	0.07	0.19°	–	
Self-Regulated Strategy Questionnaire	185.23	15.93	0.21*	0.14	0.17°	−0.07	–

***p < 0.001, *p < 0.05, °*p* = 0.06.

### Effect of Quiz and Out-of-Class Activities on Exam Performance

A two-step mixed linear model was run with R (R Core Team, 2019), using the lmerTest package (Kuznetsova et al., 2017), to analyze the effects of quiz activities (number of Top Hat sessions attended; maximum: seven) and out-of-class activities (number of activities; maximum: four) on exam performance (i.e., mean raw scores for closed and open questions without bonus points). Given that different questions were used for different exam sessions, we added exam session as a random factor to control for general differences due only to random differences in the sessions’ difficulty.

In the first step, the model was run adding only quiz activities as a predictor of exam performance. The quizzes had a significant effect (β = 0.62; *p* < 0.001) on exam results, explaining 9% of the variance (marginal). In the second step, we added out-of-class activities to the predictors of exam performance. The results showed a significant effect (*p* < 0.001) of both out-of-class activities (β = *0.77*) and quiz activities (β = *0.42*). The model explained 13% of the variance (marginal), adding 4% of variance compared with the first regression model. Given that the two predictors are not orthogonal, part of the variance explained is common between them.

**FIGURE 1:**
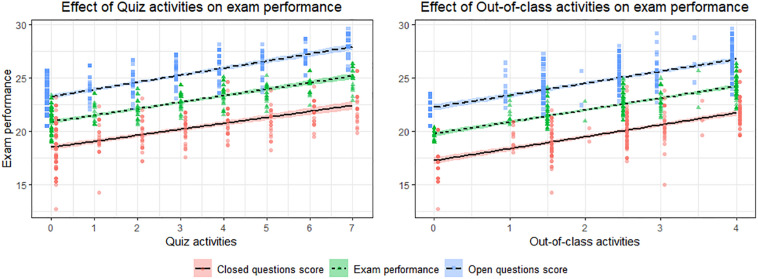
Effects of quiz activities and out-of-class activities on exam performance by question type.

To ensure a similar impact on the different types of exam question, the same analysis was run again twice, once considering the scores for the closed questions as dependent variables and once considering the scores for the open questions. The results showed a similar trend (see Figure 1). Although we were not interested in the students’ accuracy in completing the quizzes (since they were used primarily to stimulate interaction with students), its effect (the percentage of correct answers) on exam performance (total, closed, and open questions) was ascertained and the results remained comparable with those presented above.

### Relationship Between Quiz Activities, Out-of-Class Activities, Exam Performance, Theories of Intelligence, and Self-Regulated Strategy Questionnaires

We analyzed the correlations among SRSQ, TIQ, quiz, and out-of-class activities. The two questionnaires were not mandatory, and correlations for the SRSQ and TIQ could be calculated on 134 and 103 students, respectively. The results (see Table 1) showed that the quiz activities did not significantly correlate with SRSQ and TIQ scores, while the out-of-class activities showed a tendency toward significance with both the SRSQ and the TIQ scores. Exam performance significantly correlated with SRSQ score (i.e., self-regulated strategies; *r* = 0.21).

## Discussion

The results of the regression models showed that answering quizzes positively affected the final exam performance (9% of marginal variance explained). This is in line with the literature showing a positive effect on the academic performance of using SRS during lessons (Hunsu et al., 2016; Sung et al., 2016). The variance explained in the model is in line with the small effect of SRS on academic achievement in large classes (*g* = 0.10; Hunsu et al., 2016).

The effect of administering quizzes during lessons, supported by the use of an advanced SRS, can be explained by the testing effect, i.e., repeated quizzes in class make it easier to memorize the course content and foster its recall over time (Karpicke and Roediger, 2007). In the present study, the testing effect refers to the fact that being repeatedly tested on course content (through sample exam questions) during the lessons may have increased the retention of study material and support the performance during the final exam. The positive effect of quizzes may also be supported by feedback given after each answer (as in Rowland, 2014). It should be noted that this feedback was followed by class discussion to link the question to the content of the lesson. While this combination (quiz feedback and discussion) can be a useful strategy, it makes it difficult to disentangle whether and to what degree the positive effect of quizzes on exam performance was due to a specific role of feedback and/or related discussion.

The quizzes during lessons with SRS can be used to optimize the learning process by activating the students in class and enabling them to memorize what they learn more efficiently. In such contexts, where many students are grouped in the same room to hear a lecture and attendance is not mandatory, the use of technologies like SRS to administer quizzes during lessons appears to be beneficial to exam performance. Using a mobile-based technology to administer quizzes seems to have some advantages over the more traditional ways of asking questions in class (including anonymity and immediate feedback) or other technologies. It may have refined functions that enable the quizzes to be integrated with course material and make students more comfortable about following the lesson and answering questions (Kawash and Collier, 2018). Although the evidence emerging from our study cannot be generalized to mobile device use in lectures, it does support the benefits of administering quizzes, possibly *via* the students’ own mobile devices, during lessons.

Our results concerning out-of-class activities that are proposed to the students also deserve to be discussed. Additional course-related activities improved the students’ final exam performance and seemed to be just as effective as quizzes (as shown in Figure 1). The experience of preparing reports and participating in lab experiences had a positive effect on the students’ academic achievement, confirming the importance of such complementary activities (as suggested by Heng, 2014) in the learning process. A further indication of the relevance of participating in an out-of-class activity comes from the correlations with the motivational and SRSQs: significant correlations emerged not only between exam performance and self-regulated strategies (in line with previous evidence; Richardson et al., 2012; Mega et al., 2014) but also with out-of-class activities. On the other hand, completing the quiz activities did not relate to such functional motivational beliefs and self-regulated learning strategies, possibly because attending classes and completing the quizzes remain important regardless of the students’ self-reported strategies and motivational beliefs, whereas participating in out-of-class activities seemed more related to effective learning motivation and strategies. It may be that such additional activities (which demand extra time and effort) were more likely to be chosen by more motivated and strategic students. It is also plausible that only the more motivated students answered the non-mandatory questionnaires since motivation is often considered an antecedent of affective student engagement (Bond et al., 2020). As for using quizzes during lessons, this could have helped to foster active learning in class regardless of the students’ motivation by enhancing information retention and, ultimately, performance (Roediger and Butler, 2011). Although the value of out-of-class activities was not the main focus of this paper, our intriguing findings warrant further study.

More research is needed to better understand these findings and overcome some limitations of our study. Among them, there is the fact that our sample could be biased, even if the large class where attendance is not mandatory represents an ecological setting and a common condition at a university. Since attendance and involvement in the quizzes and the out-of-class activities were voluntary, it may be that only students who were already motivated took part. It is worth noting that the quizzes seemed equally appealing to students with different levels of motivation, including the more “reluctant” ones (Graham et al., 2007). Although our sample may be biased *per se* and there was no control group of other students, a *post hoc* analysis on the students who did not take part at the proposed activities showed that their average exam grades were lower than those of the students who did take part at least once. This suggests that quizzes and/or out-of-class activities can benefit performance in the final exams. Another limitation of our study concerns the fact that only two types of activating teaching method were investigated (i.e., quizzes and out-of-class activities) among the many available. In addition, the use of mobile-based quizzes is just one of a variety of applications for mobile devices in class (although it is one of the most advanced), so our results cannot be extended to their use for teaching in general.

As a final consideration, proposing quizzes and out-of-class activities seemed to promote class attendance as around 64% of students attended the lessons (registering with the Top Hat system) and took part in the out-of-class activities—a satisfactory proportion for a course for which attendance is not mandatory. This percentage is similar to that of other psychology courses (on average 65%) that also incentivize attendance. To some degree at least, this could be seen as an objective indicator of behavioral engagement (Ribeiro et al., 2019; Bond et al., 2020). Previous studies also found SRS use and out-of-class activities to be positively associated with engagement (Heng, 2014; Bond et al., 2020) and, in turn, with achievement (Lei et al., 2018). The use of SRS may promote active engagement with learning material, thereby indirectly influencing achievement (Blasco-Arcas et al., 2013; Hunsu et al., 2016). Whether and how quizzes and additional course-related activities encourage engagement through active participation deserves to be systematically investigated to shed light on the role of individual characteristics (such as motivational/strategic approach), technology use (i.e., SRS), and different types of additional activity in promoting various types of student engagement (not only behavioral but also cognitive and affective; Bond et al., 2020).

## Conclusion

Our study contributes to expanding what is known about the use of different teaching methods in higher education systems with large cohorts of students and voluntary attendance, as in this Italian experience included in this Special Issue. Although more systematic evidence will be needed, these results suggest that using technology to improve learning in class, based on quizzes during lessons and out-of-class activities, has a positive effect on the students’ exam performance. These results suggest that a combination of activating teaching modalities such as those used in the present study can be a feasible way to foster academic achievement in higher education.

## Data Availability Statement

The datasets presented in this study can be found in online repositories: doi: 10.6084/m9.figshare.11984361.

## Ethics Statement

The studies involving human participants were reviewed and approved by the Ethics Committee for Research in Psychology (University of Padova). The patients/participants provided their written informed consent to participate in this study.

## Author Contributions

DA and CD provided the technical support on the use of Top Hat/SRS. TF performed the statistical analysis. TF and CT organized the database. NC wrote the first draft of the manuscript. TF, CM, and CT contributed to the sections of the manuscript. All authors contributed to the conception and the design of the study, contributed to revising the manuscript, and have read and approved the submitted version.

## Conflict of Interest

TF was employed by the company Pentathlon Srl. The remaining authors declare that the research was conducted in the absence of any commercial or financial relationships that could be construed as a potential conflict of interest.

## Footnotes

1 https://tophat.com/
